# Comparison of blood lactate and perceived exertion responses in two matched time-under-tension protocols

**DOI:** 10.1371/journal.pone.0227640

**Published:** 2020-01-15

**Authors:** Salvador Vargas-Molina, Fernando Martín-Rivera, Diego A. Bonilla, Jorge L. Petro, Leandro Carbone, Ramón Romance, Manuel deDiego, Brad J. Schoenfeld, Javier Benítez-Porres

**Affiliations:** 1 EADE-University of Wales Trinity Saint David, Málaga, Spain; 2 Human Kinetics and Body Composition Laboratory, University of Málaga, Málaga, Spain; 3 Research Unit in Sports and Health, University of Valencia, Valencia, Spain; 4 Research Division, DBSS International, Bogotá, Colombia; 5 Research Group in Physical Activity, Sports and Health Sciences (GICAFS), Universidad de Córdoba, Montería, Colombia; 6 University of Salvador, Buenos Aires, Argentina; 7 Health Sciences Department, CUNY Lehman College, Bronx, NY, United States of America; Ritsumeikan University, JAPAN

## Abstract

**Purpose:**

The aim of this study was to compare the concentration of blood lactate [bLa-] and the subjective perception of exertion of trained men in a moderate repetition protocol (MRP) versus a high repetition protocol (HRP) equated for time under tension.

**Methods:**

A sample of 40 healthy young men (aged, 23.2 ± 4.0 years; height, 177.3 ± 7.0 cm; BMI, 24.3 ± 2.2) performed two sessions of 8 sets of bicep curls with a one-week recovery interval between the trials. In the HRP protocol, 20 repetitions were performed with a cadence of 2 seconds of eccentric and 1 second of concentric, while in the MRP protocol 10 repetitions were performed with 4 seconds of eccentric and 2 seconds of concentric. Cadences were controlled by a metronome. At the beginning and end of each of the sessions, blood lactate was taken at 2, 15, and 30 minutes, and rating of perceived exertion (OMNI-RES) was assessed immediately after completion of each session.

**Results:**

There were [bLa-] differences between protocols in the MRP 2 min, (5.2 ±1.4); 15 min, (3.2 ±1.2); 30 min, (1.9 ±0.6); p< 0.05, and the HRP 2 min, (6.1 ±1.6); 15 min, (3.7 ±1.1); 30 min, (2.2 ±0.6); p<0.01. OMNI-RES was higher in HRP, (8.8 ±0.7) than in MRP, (7.7 ±0.9). Additionally, a correlation was found between the RPE and [bLa-] values in the HRP protocol (rs = 0.35, *p* < 0.01).

**Conclusions:**

Training protocols with high times under tension promote substantial increases in metabolic stress, however, our findings indicate that HRP generates more [bLa-] than MRP. In addition, there were higher RPE values in the HRP protocol compared to MRP in single-joint exercises.

## Introduction

Mechanical tension (MT) has been identified as a critical factor for eliciting a stimulus necessary to activate molecular signaling pathways related to muscle protein synthesis (MPS) and, consequently, to enhance skeletal muscle development [1]. MT is mainly dependent on two variables. The first is the load used, which is proportional to the tension generated. The second is the time under tension (TUT), which indicates the need to maintain a certain load for a minimum amount of time that optimizes the relationship between the generated tension and work volume [2].

However, MT does not seem to be the only mechanism involved in eliciting a MPS response; metabolic stress (MS) produced during resistance training (RT) is also theorized to play a role [3]. From an energy standpoint, RT strategies for the development of muscle mass rely largely on the glycolytic pathway, which in turn generates an accumulation of metabolites–particularly lactate, inorganic phosphate and H^+^ [3].

The concentration of blood lactate [bLa^-^] depends on several factors including the volume and relative intensity of work, the amount and size of the muscle mass involved in the exercise [4], and the load and speed of execution [5]. Research indicates [bLa^-^] and changes in pH are associated with the acute hormonal responses that are evident after RT. These acute hormonal elevations have been proposed to play a role in RT-induced muscle hypertrophy [6, 7], although emerging evidence refutes such claims [8]. Thus, the measurement of [bLa^-^] constitutes an indicator of MS in RT [9]. The change in the perception of this metabolite from being a waste molecule derived from pyruvate reduction to one involved in different adaptations to exercise poses new perspectives for research. Specifically, research indicates a positive relationship between [bLa^-^] and muscle protein anabolism, with results showing enhanced differentiation of satellite cells, an increase in myogenic protein content, and greater phosphorylation of ribosomal protein S6 kinase beta-1 (RPS6KB1, also known as P70^S6K^) [10]. Recently, Tsukamoto et al. [11] demonstrated that intraperitoneal injection of lactate in mice elicited a hypertrophic response compared to controls; importantly, lactate levels were similar to those induced by moderate repletion RT protocols, suggesting a potential physiological role. Many molecular mechanisms involved in this process are currently unknown but may be related to the relationship between [bLa^-^] and muscle anabolism; therefore, the mechanisms behind these adaptations have not yet been sufficiently identified, and further research is needed to support this relationship [12]. Notably, metabolite accumulation alone does not seem sufficient to substantially increase muscle mass in humans; however, it may have a positive additive effect on anabolic processes due to its capacity to generate an increase in muscle activation [13], promote the recruitment of high-threshold motor units [14], and trigger the production of myokines and reactive oxygen species that have been implicated in hypertrophic adaptations [3].

To elicit greater MS, the TUT can be manipulated via the cadence or the number of repetitions. In both strategies, [bLa^-^] seems to be a reliable marker of MS for the above-mentioned reasons, but this relationship is not entirely linear when comparing different strategies [4]. Moreover, when applying advanced techniques such as supersets and tri-sets, greater increases in [bLa^-^] are observed compared to traditional protocols [15].

In addition, the subjective rating of perceived exertion (RPE) has been shown to be an effective tool for assessing both load and fatigue during resistance exercise [16–20]. Kraemer et al., demonstrated increases in [bLa^-^] as well as RPE after 3 sets of 4 resistance exercises performed at a load corresponding to a 10-repetition maximum (10-RM) [21]. Several studies have reported a high correlation between the load increase and the subsequent rise in the RPE response [18–20, 22, 23]. Moreover, both muscle activation, as estimated by electromyographic activity, and time under tension have been shown to be directly related to the perceived exertion response [19, 22]. It appears that metabolite accumulation can affect afferent feedback in the central nervous system and thus decrease performance and contractile efficiency [24]. Moreover, this may in turn influence RPE. However, the exact mechanisms for these findings is as yet undetermined.

The purpose of the present study was to evaluate [bLa^-^] after two different training protocols: one with high repetitions (HRP) and the other with moderate repetitions (MRP), using different cadences in the muscle actions (concentric and eccentric) but with an equated TUT (60 seconds). Additionally, the RPE was evaluated at the end of each protocol with the OMNI-Resistance Exercise Scale (OMNI-RES), validated by Robertson et al. [16], to evaluate the load intensity and, secondarily, to determine the correlation between [bLa^-^] and RPE in load protocols that induce MS.

## Material & methods

### Sample

The sample size was calculated by G*Power 3.1 using the following criteria: effect size f(V) = 0.71 (Cohen et al., 1988), α err prob = 0.05, Power (1-β err prob) = 0.95. It was determined that 36 subjects were needed for analysis. To account for potential dropouts, a total of 40 volunteers with more than 1.5 years of consecutive experience in RT participated in the present study (age = 23.2 ± 4.0 years; height = 177.3 ± 6.9 cm; body mass = 76.4 ± 7.8 kg; BMI = 24.3 ± 2.2 kg∙m^2^). A crossover design of repeated measures was used, whereby all subjects performed each of the training protocols across 4 sessions, with each session separated by 7 days for recovery.

Subjects who reported using doping substances (e.g., anabolic-androgenic steroids) during the last two years and/or who consumed any type of dietary supplement during the program were excluded from participation. The following restrictions were imposed on volunteers: no food, drinks, or stimulants (e.g., caffeine) to be consumed 3–4 hours before the sessions and no physical activity more intense than daily activities 12 hours before the exercises. Moreover, subjects were instructed to sleep at least 8 hours the night before data collection, have breakfast at least two hours before the tests, and to avoid stimulants such as coffee. Subjects were advised of the potential risks of the experiment and signed an informed consent form. The research protocol was reviewed and approved by the Ethics Committee of the University of Málaga (code: 38-2019-H). The study was developed in accordance with the ethical guidelines of the Declaration of Helsinki [25].

### Procedures

#### Familiarization sessions

Before the intervention, 4 sessions were carried out with 7 days of recovery between each session, 2 for familiarization and 2 for each protocol (HRP and MRP); all sessions were, supervised by the principal investigator. Once each familiarization session was finished, the participants were instructed not to perform any resistance training involving the elbow flexors over the 48 hours prior to the ensuing testing session.

The familiarization sessions began with a warm-up consisting of 4 sets of 10 repetitions at a load that ranged between 30 and 40% of the estimated repetition maximum (RM). Between 3 to 5 tests were performed with each protocol to estimate individual loads while using the metronome to control cadence, with a 5-minute rest interval afforded between each test. Repetitions were completed throughout a full range of motion in elbow flexion-extension. Subjects were also instructed to identify their RPE using OMNI-RES.

#### Experimental sessions and evaluation of study variables

The participants (n = 40) performed two protocols over two sessions separated by 7 days with the goal of reaching concentric failure on each set. The first protocol was the HRP, which consisted of 8 sets of approximately 20 repetitions of biceps curls with a 1-minute rest interval between sets. Each repetition had a duration of 3 seconds (2 seconds afforded for the concentric action and 1 second for the eccentric action). The second protocol was the MRP, which involved the same exercise, rest interval, and number of sets as the HRP, but with approximately 10 repetitions per set and a tempo of 4 seconds for the eccentric action and 2 seconds for the concentric action. Both protocols had a similar total TUT of ~60 seconds per set (Table 1). A metronome (App Metronome M1) was used to control the duration of each concentric and eccentric muscle action; tempo was adjusted to 60 rpm with a ^2^/_4_ beat in the MRP and ¾ beat in the HRP.

**Table 1:** Intervention protocols.

Protocols	Sets	Repetitions	AE	AC	TUT sets	TUT AE TS	TUT AC TS	TUT TS
MRP	8	~10	4	2	60 s	320 s	160 s	480 s
HRP	8	~20	2	1	60 s	320 s	160 s	480 s

MRP, moderate repetitions protocol; HRP, high repetitions protocol; AE, eccentric muscle activations; AC, concentric muscle activation; TUT, time under tension; TS, set total; s, seconds.

The experimental sessions began with an initial measurement of [bLa^-^], which was obtained after subjects relaxed in a seated position for 10 minutes. Subjects then performed a warm-up, which consisted of 4 sets of 10–12 repetitions at 30–40% RM, utilizing the corresponding cadences for each respective testing protocol. The load lifted at the beginning of the training sessions was obtained from the familiarization sessions. As fatigue occurred, the loads were accordingly reduced when the target number of repetitions and/or TUT established by the protocol was not reached. In addition, if the subjects lost the rhythm established by the metronome and finished the target repetitions prior to achieving the 60-second TUT, they were instructed to continue training until the target TUT was attained. The mean ± SD of the daily training loads that the participants used for the HRP and MRP protocols were 20.69 ± 2.12 kg and 20.36 ±1.7 kg, respectively.

The protocols, with their respective familiarization sessions, were conducted in a counterbalanced manner. The first subject was randomly assigned to either the HRP or MRP training, and thereafter the protocols were counterbalanced. Thus, half of the subjects performed MRP in the first experimental session while the other half performed HRP; each subject then performed the alternative protocol in the second session.

**Fig 1:**
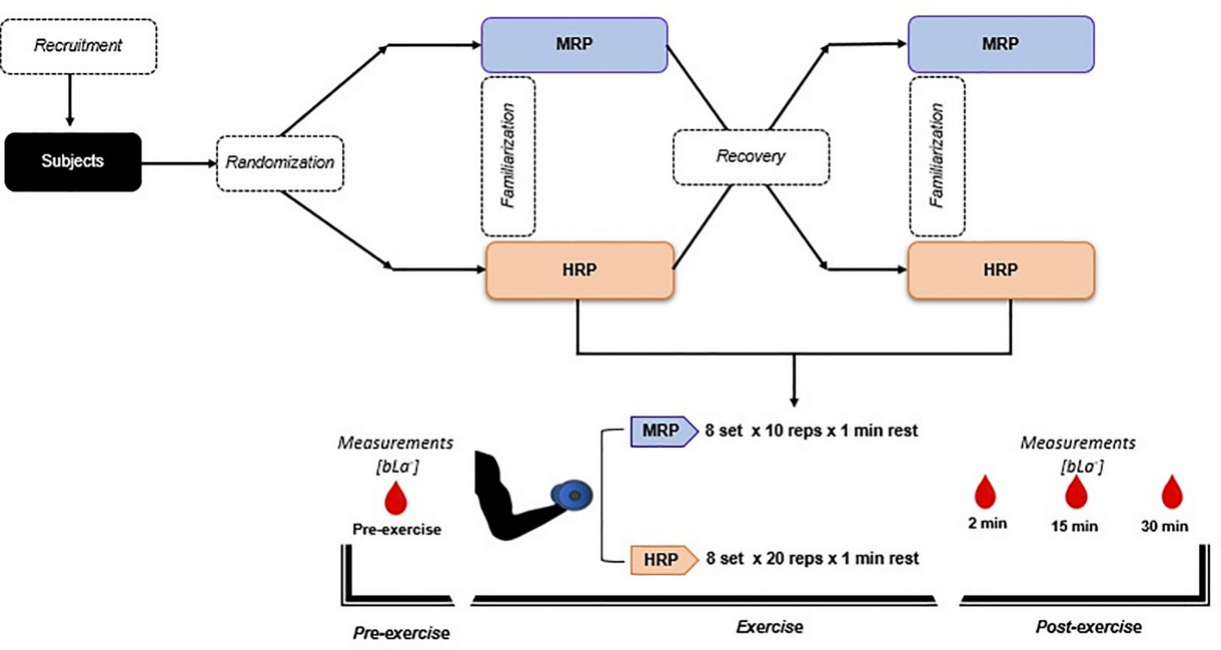
Experimental design.

Following completion of each respective 8 set testing protocol, blood samples were taken for the measurement of [bLa^-^] at 2, 15, and 30 minutes (Fig 1). Collection of blood was carried out with subjects relaxed in a sitting position. Samples were obtained from the ear lobe, a conventional sampling site [26, 27], after the lobe was cleansed and sterilized with 70% ethanol. A ≥ 0.5 μl sample of blood was collected and then treated with a lactate analyzer (Lactate Scout+, SensLab GmbH, Leipzig, Germany).

Additionally, we obtained RPE values on a scale of 1–10, as described by Robertson et al. [16]. Specifically, subjects were instructed to report their perceived exertion immediately at the end of the 8 series of each protocol. They were told that numerical value 2 corresponded with easy, 3–4 somewhat easy, 6 somewhat hard, 8–9 hard and 10 extremely hard.

### Statistical analysis

All results were expressed as mean (M) and standard deviation (SD). All variables met the normality assumption (e.g. Shapiro-Wilk), except for RPE whose values were not normally distributed. To compare the influence of the type of protocol used in relation to lactate concentration, an analysis of the variance of repeated measurements was used. The protocol variable had two levels (MRP, HRP) and the lactate concentration [bLa^-^] had a total of four levels (pre, 2 min, 15 min, 30 min). In the case of non-compliance with Mauchly´s sphericity assumption, the Greenhouse-Geisser correction was performed. In all analyses that were significant in the ANOVA omni-bus, Bonferroni’s post Hoc was performed. The size of the effect was calculated using Cohen’s d. To compare the influence of the type of protocol used in relation with RPE, the Wilcoxon test for two related samples was performed. The Spearman test was applied to stablish the correlation between the RPE and lactate concentration [bLa^-^] at 2 minutes post-exertion. P values < 0.05 were considered statistically significant. Statistical analyses were performed with licensed Statistical Package for the Social Sciences software (SPSS 24.0, IBM Corp., Armonk, N.Y., USA) and GraphPad Prism software version 7.03 (GraphPad software, California, USA).

## Results

The results showed a significant interaction between protocol and time of sampling factors F(1.82, 71.303) = 4.45, p<0.05 (0.018), η_p_^2^ = 0.102 in the amount of lactate obtained in blood [bLa^-^]. Peer comparisons, according to the intervention protocol performed, showed statistically significant differences according to the intervention protocol performed in bLa2, bLa15, bLa30 with higher lactate concentrations noted in the HRP protocol. No difference was found between the two protocols in the resting [bLa^-^].

**Fig 2:**
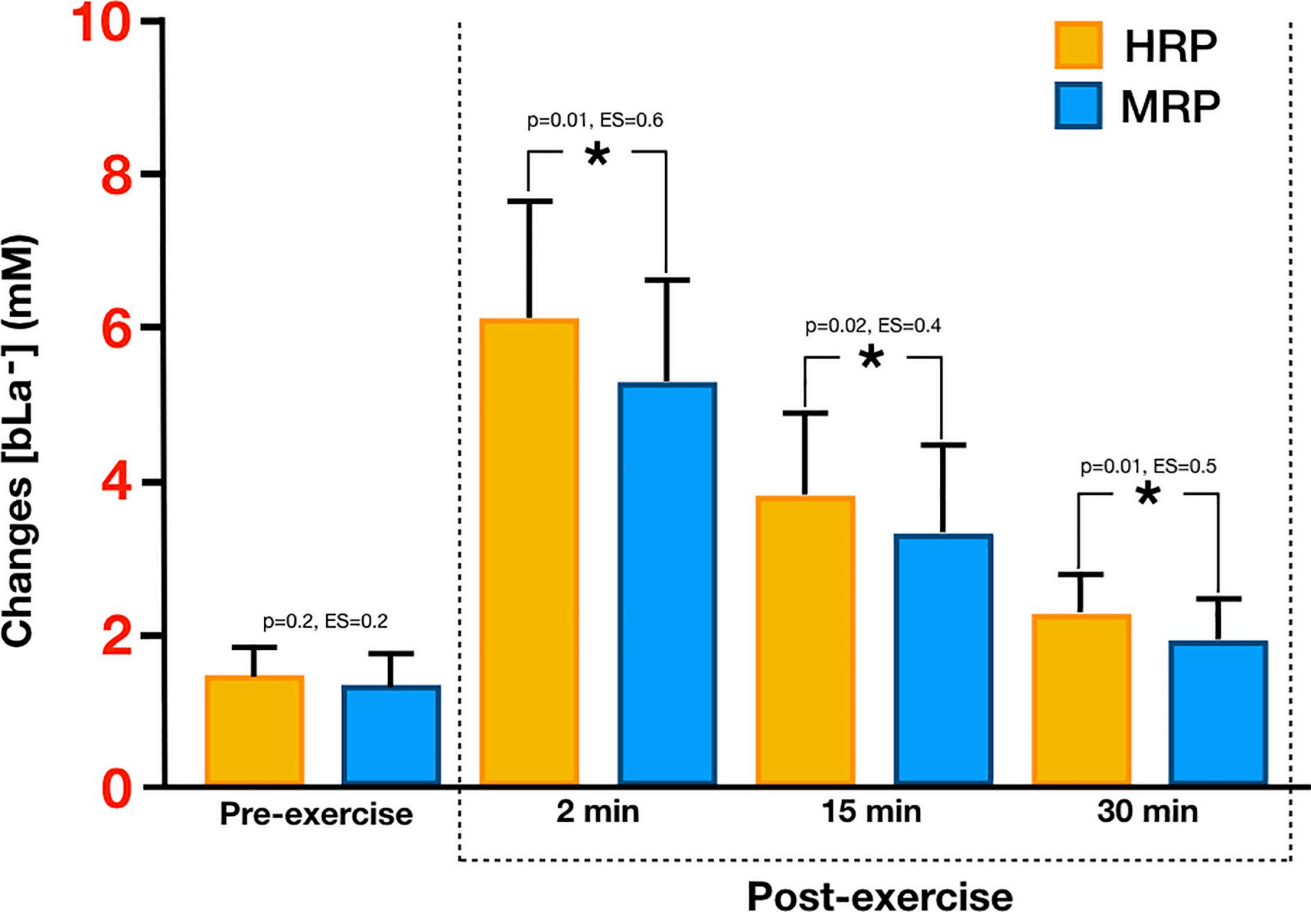
Changes in blood lactate concentrations.

The *post hoc* analysis showed differences between each [bLa^-^] sampling time for both protocols (p = 0.015). Regarding the comparison of means between the protocols, the [bLa^-^] values evaluated at 2, 15, and 30 minutes post-exercise were significantly higher (*p* < 0.05) in the HRP protocol for all timepoints. The changes in the concentration of [bLa^-^] confirm the significant differences for both protocols relative to the initial values, with differences observed between the two protocols (Fig 2).

**Fig 3:**
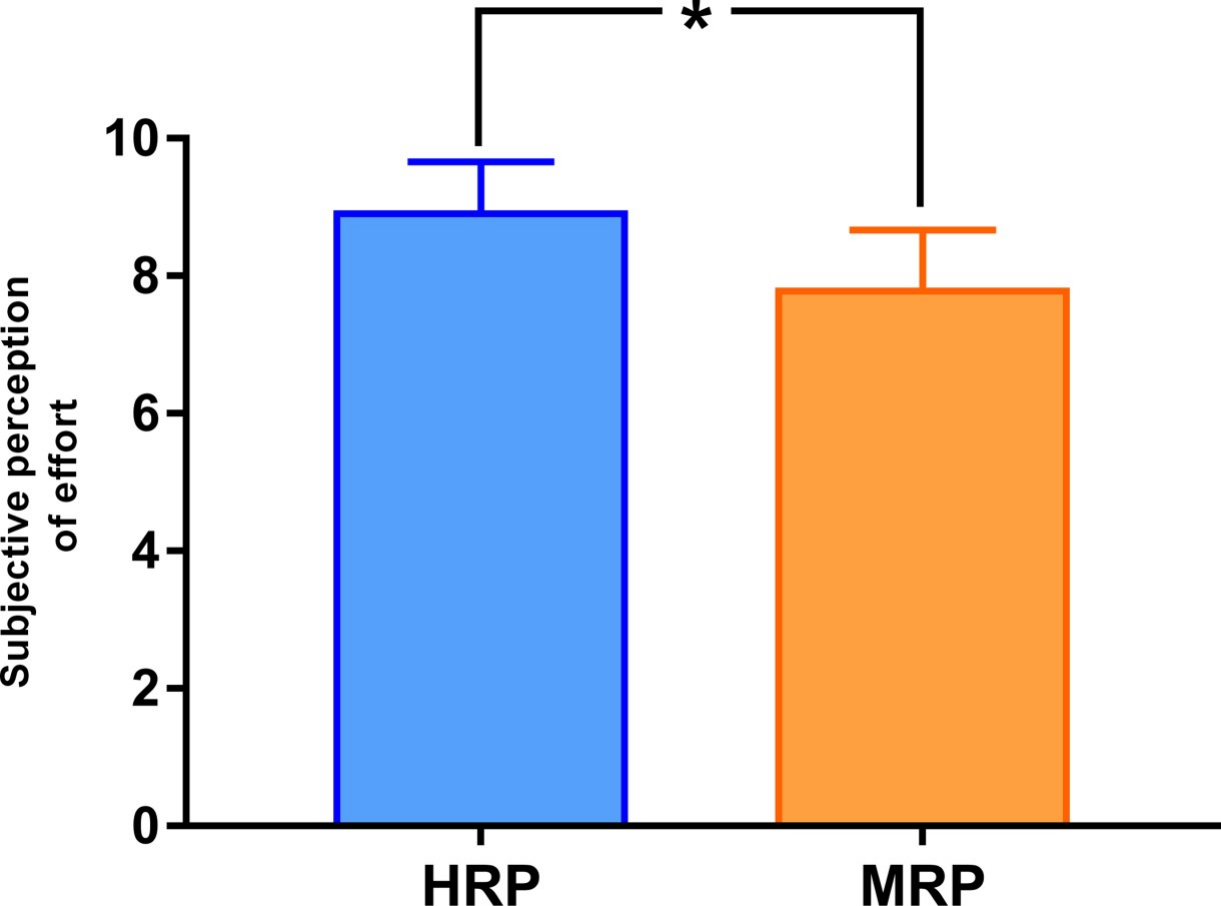
Perception of exertion in high repetitions protocol and moderate repetitions protocol.

Regarding the RPE, a significantly greater (*p* < 0.05) perception of exertion (8.8 ± 0.7) was reported for the HRP compared to the MRP (7.7 ± 0.9), ES = 1.4 (Fig 3). According to the scale used, values equal to or greater than 8 are considered difficult, which shows the high level of exertion of both protocols, especially the HRP protocol.

**Fig 4:**
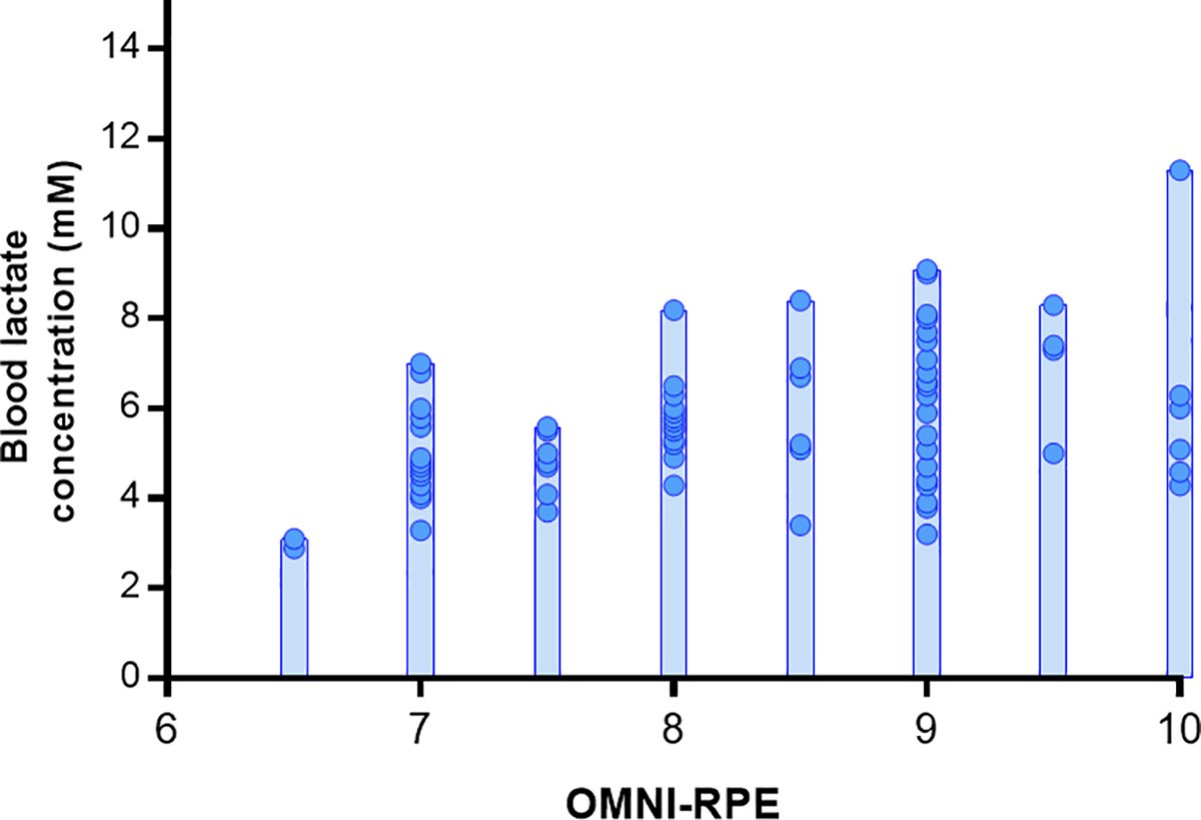
Dispersion of lactate values at two minutes and subjective perception of exertion.

In addition, a significant correlation was found (rs = 0.35, *p* < 0.01) between the [bLa^-^] at 2 minutes after physical exertion and the RPE. The dispersion of the data of these two load indicators is shown in Fig 4.

## Discussion

The effect of RT-induced MS on body composition, particularly on lean body mass (LBM), has been investigated using protocols with a high TUT involving a high number of repetitions (20–25 repetitions per set) [28], giving rise to total TUTs that can range between 75 and 105 seconds per set [2]. Considering that the objective of these RT protocols is to generate greater MS using a smaller proportion of MT, it can be hypothesized that performance of a greater number of repetitions and thus more muscle actions (concentric–eccentric cycle) per set would be beneficial in this regard [1].

The results of the present study showed large, significant differences in the post-exercise [bLa^-^] response between HRP and MRP; although both protocols elicited high levels of MS, elevations in [bLa^-^] were significantly greater in the HRP protocol. Our results agree with the findings of Lacerda et al. [29], who observed higher [bLa^-^] in a RT protocol in which more repetitions were performed. A notable difference between studies was that Lacerda et al. [29] employed a TUT of 36 seconds whereas the TUT was markedly higher in the present study (60 seconds). The totality of findings suggest that repetition range plays the dominant role in post-exercise MS as opposed to the speed at which repetitions are performed.

It is important to note that elevations in [bLa^-^] are context-specific. This is highlighted in the discrepant results in studies from the lab of Gentil et al. An initial study [4] showed differences in [bLa^-^] with a protocol executed at low speed lasting 60 seconds compared to three other conventional protocols (10 RM, functional isometrics with 5 seconds of isometric contraction at 90º of elbow flexion, and venous vascular occlusion). The protocol that obtained the smallest increase in [bLa^-^] was the one executed at the lowest speed; however, the 60 second TUT in this protocol constituted a single repetition (30 seconds for both concentric and eccentric actions). Thus, the study design differed substantially from the present study. A follow-up study [30] evaluated the same protocols as investigated previously [4], but also included forced repetitions, descending sets, and 6 RMs. In this instance, results showed similar elevations in [bLa^-^] between protocols, including the one performed at a very slow speed (with 60 seconds in a single repetition).

A practical consideration is our finding of a higher RPE in the HRP protocol, with a correlation observed between RPE and [bLa^-^]. This finding is consistent with that of Aniceto et al. [18], who reported the same correlation in strength-trained subjects during performance of a circuit training protocol. From a practical application standpoint, this approach therefore conceivably can be used to manipulate training intensity without the need for invasive testing procedures. It therefore is reasonable to speculate that RPE can be used to gauge RT-induced MS irrespective of whether results are due to a slower cadence or a higher number of repetitions.

Although our study provides intriguing insights into the metabolic responses to different training protocols, it is important to point out that findings are specific to acute RT performance and do not reflect how such results may translate into long-term adaptations. Moreover, findings are specific to a young, resistance-trained individuals and thus cannot necessarily be generalized to other populations. In addition, findings are specific to performance of a sole, single-joint exercise, and thus cannot be extrapolated to performing multiple compound exercises, as is often the case in ecologically valid RT programs. Further, despite our attempts to ensure failure occurred precisely within the target TUT, individual variation did not always allow this to occur in practice. Thus, while all subjects trained with a high degree of effort (if not reaching failure, then stopping within 1 to 2 repetitions of failure), we cannot rule out the possibility that achieving absolute failure in all subjects across all sets may have differentially influenced results. Finally, the overall magnitude of lactate responses was fairly modest, and thus the ramifications on how such moderate elevations may play a role in muscular adaptations is not clear.

## Conclusions

The HRP generated significantly greater increases in post-exercise [bLa^-^] compared to MRP. In addition, RPE was higher in the HRP compared to the MRP, and RPE was found to correlate with the extent of [bLa^-^]. In view of these findings, prolonging repetition cadence does not seem to be a suitable option when the aim is to achieve higher levels of MS.
